# Phosphite Compounds Suppress Anthracnose in Soybean Seeds Infected by *Colletotrichum truncatum* and Stimulate Growth and Defense Mechanisms

**DOI:** 10.3390/plants14101494

**Published:** 2025-05-16

**Authors:** Manoel Batista da Silva Júnior, Mário Lúcio Vilela de Resende, Edson Ampélio Pozza, Alexandre Ribeiro Maia de Resende, Gustavo César Dias Silveira, Jayne Deboni da Veiga, Júlia Marques Oliveira, André Costa da Silva

**Affiliations:** 1 Departamento de Fitopatologia, Universidade Federal de Lavras, Lavras 37200-000, Minas Gerais, Brazil; mjunior_agroufla@yahoo.com.br (M.B.d.S.J.); mlucio@ufla.br (M.L.V.d.R.); eapozza@ufla.br (E.A.P.); alexandrermresende@yahoo.com.br (A.R.M.d.R.); gcsagro@gmail.com (G.C.D.S.); 2 Departamento de Fitossanidade, Universidade Federal do Rio Grande do Sul, Porto Alegre 91540-000, Rio Grande do Sul, Brazil; jaynedeboniveiga73@gmail.com; 3 Centro Universitário de Formiga, Formiga 35574-530, Minas Gerais, Brazil

**Keywords:** *Glycine max* L., seed treatment, potassium phosphite, zinc phosphite, manganese phosphite, copper phosphite

## Abstract

Soybean is one of the main agricultural commodities, and its productivity is limited by several diseases, such as anthracnose, which is caused by a complex of fungal species, with *Colletotrichum truncatum* being the most prevalent. Management is mainly carried out through chemical seed treatment. However, a reduction in the sensitivity of *C. truncatum* to fungicides was observed. Therefore, it is extremely important to search for products that are effective in controlling the disease. The objectives of this study were to evaluate the efficacy of commercial formulations of copper, potassium, manganese, and zinc phosphites in the treatment of soybean seeds infected by *C. truncatum*, as well as their direct fungitoxicity and ability to induce soybean defense mechanisms. For this purpose, seeds inoculated with *C. truncatum* were subjected to phosphites and a fungicide (carbendazim + thiram). The seeds were exposed to germination, health, and vigor tests. Fungal toxicity and the ability of phosphites to induce defense through the activities of catalase, peroxidase, and superoxide dismutase enzymes, as well as the levels of lignin and total soluble phenols, were also evaluated. Mn and Zn phosphites showed direct toxicity to *C. truncatum* and were as effective as the fungicide (carbendazim + thiram) in treating soybean seeds infected by the fungus. Mn phosphite induced the production of catalase (CAT), peroxidase (POX) and lignin, while Zn phosphite increased the production of CAT and POX. These results demonstrate the efficacy of Mn and Zn phosphites in controlling *C. truncatum* in infected soybean seeds, their direct toxic action, and their ability to induce resistance.

## 1. Introduction

Soybean (*Glycine max* [L.] Merrill) is one of the most important commodities in the world [1]. Rich in proteins (40%) and moderate in oil (20%), it is used in both human and animal feed [2] and is of vital importance for ensuring global food security [3]. However, the crop can be affected by various pathogens from seed germination to grain filling. Anthracnose is one of the most significant fungal diseases affecting soybean [4] and is prevalent in nearly all soybean-growing areas worldwide, causing yield losses that can reach up to 100% [5,6]. Approximately 90 kg/ha of grain yield is lost for each 1% increase in disease incidence in commercial soybean fields [7]. Although new species have recently been reported to be associated with anthracnose, *Colletotrichum truncatum* (Schw.) Andrus & WD Moore is the predominant species in soybean fields [4,8]. *C. truncatum* is one of the most common and harmful seed-borne pathogens affecting soybean, particularly when the crop is grown under warm and humid conditions [9]. The fungus can attack all parts of the plant at all developmental stages [10,11]. In infected seeds, it causes a significant reduction in seed quality, leading to a decrease in plant stand [12]. Typical anthracnose symptoms include pre- and post-emergence damping-off; dark, sunken, and irregular lesions on cotyledons, stems, petioles, and pods, where acervuli are observed [8]; immature pod opening, resulting in seed germination [13]; and necrotic lesions on the abaxial leaf veins, which can lead to premature defoliation [5,8]. Recurring reports of severe epidemics and significant productivity losses have increased each year [8]. Management of this disease is carried out through seed chemical treatment or genetic resistance [14,15]. However, highly resistant cultivars are not known in Brazil. Chemical control remains the most commonly used method for this purpose. However, studies have demonstrated a reduction in *C. truncatum* sensitivity to fungicides recommended for the crop [7,16,17]. *C. truncatum* exhibits high genetic variability [8], which allows the fungus to adapt to various environmental conditions, hosts, and control measures implemented in its management. This high variability complicates genetic improvement efforts in the search for soybean cultivars with durable resistance and results in a reduction in the fungus’s sensitivity to fungicides used for its control. Moreover, due to the harmful effects of pesticides on the environment and human health, as well as a reduction in *Bradyrhizobium* viability and consequent damage to nodulation [18], new alternative seed treatment methods for controlling anthracnose in soybeans are necessary. Among the alternative products available on the market, phosphites stand out. These products are obtained through the reaction of phosphorous acid with a base (K^+^, Ca^2+^, among others). They can act on nutrition, direct toxicity to the pathogen, and induce resistance in the host [19]. Another important feature of phosphites is their low cost compared to chemical fungicides [20]. Phosphite-based compounds have been widely studied for their efficacy in managing various plant diseases [21,22], and numerous studies have demonstrated their potential as alternatives to conventional fungicides. They have been mainly used in the management of foliar diseases in different crops [23,24,25], including soybeans [26]. Some studies have also shown their effectiveness in seed treatment, such as in cucumber seeds for controlling *Pythium* spp. [27], in hemp seeds against damping-off [28], in potato seeds for controlling *Fusarium solani*, *Rhizoctonia solani*, and *Phytophthora infestans* [29], and in soybean seeds for controlling *Macrophomina phaseolina* [30] and *Pythium* spp. [21]. In addition, phosphites have shown low phytotoxicity [21,27], compatibility with biological control agents [30], and potential to stimulate plant growth [26,31]. Their systemic translocation via both xylem and phloem allows for local and systemic protection within the plant [32,33,34], making them suitable for various application methods, including foliar sprays, soil drenches, and seed treatments. Thus far, no studies have been found regarding soybean seed treatment with phosphites for the control of anthracnose, and few studies have tested phosphites in seed treatment. Given the increasing importance of anthracnose worldwide and reports of *C. truncatum* resistance to fungicides recommended for the crop, this study aimed to evaluate the efficacy of commercial formulations of copper, potassium, manganese, and zinc phosphites in the treatment of soybean seeds infected by *C. truncatum*, their direct fungitoxicity, and ability to induce soybean defense mechanisms.

## 2. Materials and Methods

### 2.1. Fungal Strain

The isolate of *Colletotrichum truncatum* LAPS-473, obtained from the Seed Analysis and Pathology Laboratory Collection at the Federal University of Lavras (UFLA), Lavras, Brazil, was used. Fragments of the fungus were placed at the center of a Petri dish containing Potato-Dextrose-Agar (PDA) at 4%. The Petri dishes were then kept in a growth chamber at 27 ± 1 °C with a 12 h photoperiod at 40 μmol s^−1^ m^−2^ for 7 days. A monosporic culture was obtained from the colony, following the method described by Magalhães et al. [35], and was used throughout the experiment.

### 2.2. Sources and Doses of Phosphites and Fungicide

Four commercial phosphite formulations (Phi) obtained from Agrichem (Ribeirão Preto, São Paulo, Brazil) were tested: Reforce Mn^®^ (MnPhi; 10% Mn + 51.5% P_2_O_5_), Reforce Zn^®^ (ZnPhi; 10% Zn + 34% P_2_O_5_), Yantra^®^ (KPhi; 29% K_2_O + 33.6% P_2_O_5_), and Reforce Cu^®^ (CuPhi; 4% Cu + 20.3% P_2_O_5_) at doses of 500, 500, 500, and 250 mL.100 kg^−1^ of seeds, respectively. The commercial fungicide Protreat^®^ (150 g of carbendazim.L^−1^ + 350 g of thiram.L^−1^) from Novozymes BioAq Produtos para Agricultura Ltd. (Quatro Barras, Paraná, Brazil), recommended for seed treatment to control anthracnose, was used as a positive control treatment at a dose of 200 mL.100 kg^−1^ of seeds.

### 2.3. Inoculation of Soybean Seeds with C. truncatum

Soybean seeds of the TMG 1176 RR cultivar, highly susceptible to the fungus *C. truncatum*, were obtained from the Mato Grosso Foundation. Initially, the seeds were subjected to germination and health tests to determine the quality of the seed lot, following the Rules for Seed Analysis [36]. Subsequently, the seeds were inoculated using the water restriction method [37]. For this purpose, the PDA culture medium was prepared at an osmotic potential of −1.0 MPa by adding mannitol (80 g/L), which acts as a water restrictor to prevent seed imbibition and premature germination. Ten milliliter portions of the sterilized PDA medium were transferred to 90 mm Petri dishes and allowed to solidify. Three 5 mm diameter disks containing *C. truncatum* mycelium were placed at the center of each Petri dish and incubated in a growth chamber at 27 ± 1 °C [12] with a photoperiod of 12 h at 40 μmol s^−1^ m^−2^ until the fungus grew uniformly across the plate. For the inoculation process, 400 seeds per treatment were used, divided into 8 replicates of 50 seeds each. The seeds were carefully placed in contact with the mycelium and incubated for 28 h under the same conditions to ensure uniform exposure. Due to space limitations, the initially described setup of 100 seeds per 90 mm Petri dish was adjusted to avoid overcrowding and ensure effective inoculation. Following inoculation, different evaluation methods were applied to assess seed health and germination. The seed health test (blotter test) was conducted using 150 mm acrylic plates, with 50 seeds per plate and a total of 8 replicates. The germination test was performed using the roll paper method (28 × 38 cm sheets), also with 8 replicates of 50 seeds each. The emergence test was conducted in sand trays (40 cm length × 30 cm width × 12 cm depth) under controlled conditions. After the incubation period, seeds were removed and dried in a laminar flow cabinet for 24 h to eliminate any residual moisture absorbed during the process. This step ensured that the seeds remained viable and did not germinate prematurely before being subjected to further evaluations.

### 2.4. Seed Treatments

Soybean seeds inoculated with *C. truncatum* were subjected to the treatments described above. The seeds were placed in 1 L plastic bags with the treatments and manually agitated for 2 min. Then, the bags were kept open for drying and for the treatments to adhere to the seeds. The control treatment consisted of inoculated seeds that were not treated, and non-inoculated seeds treated with water.

### 2.5. Effects of Phosphites on Seed Health and Germination

The standard germination test (roll paper method) and blotter test, described in the manual issued by MAPA [36], were employed to determine the incidence of *C. truncatum* and the percentage of germinated seedlings that emerged from inoculated treated seeds, inoculated non-treated seeds, and non-inoculated non-treated seeds, as well as their health quality. A total of 400 seeds per treatment were used, divided into eight replicates, each containing 50 seeds. For the standard germination test, the seeds were incubated in a growth chamber at 25 ± 1 °C with a 12 h photoperiod at 40 μmol s^−1^ m^−2^ for 7 days. For the blotter test, seeds were subsequently maintained at 20 °C under a 12:12 h photoperiod for 8 days and then examined individually under a stereomicroscope to determine the level of *C. truncatum* infection. The experiment was conducted in a completely randomized design.

### 2.6. Emergence and Development of Soybean Seedlings Under Greenhouse Conditions

The seeds were sown in trays containing sand, previously autoclaved for 1 h at 121 °C and 1 atm, and were irrigated daily using a controlled micro-spray system. The emergence speed index (ESI) was determined according to the methodology described by Maguire [38]. Evaluations were conducted daily by counting the number of seedlings that emerged until the stabilization of the plant population. On the 7th and 14th day after sowing, initial (IS) and final (FS) stand establishment were assessed. On the 28th day after sowing, each plantlet was cut at the soil surface, and its height (H) and root length (R) were recorded. To determine the dry weight biomass of the aerial part (BA) and roots (BR), plantlets were transferred to paper bags and maintained in a forced air oven at 60 °C until constant weight was attained. The whole experiment was conducted according to a randomized block design with 8 treatments replicated two times with 8 plots of 50 seeds per replicate totaling 400 seeds per treatment.

### 2.7. Fungitoxic Activity of Phosphites

The fungicide carbendazim + thiram, MnPhi, and ZnPhi were tested for their efficacy in controlling anthracnose in infected soybean seeds. The fungicidal capacity of the phosphites (Phi) against *C. truncatum* was evaluated according to the methodology of Araújo et al. [39]. Discs of 5 mm in diameter, containing *C. truncatum* mycelium, were placed at the center of Petri dishes and incubated in a growth chamber at 27 ± 1 °C with a 12 h photoperiod at 40 μmol s^−1^ m^−2^ for 8 days. MnPhi and ZnPhi were tested at doses of 1.25, 2.5, 5, and 10 mL.L^−1^, and the fungicide was applied at the commercial dose (2 mL.L^−1^) to the molten PDA culture medium, which was then poured into 9 cm diameter Petri dishes. After the medium solidified, 5 mm diameter mycelial discs of *C. truncatum* were placed in the center of each Petri dish. PDA medium without any treatment was used as the control. The plates were incubated in a growth chamber at 25 ± 1 °C with a 12 h photoperiod at 40 μmol s^−1^ m^−2^. Two orthogonal measurements of colony diameter were taken at a fixed time each day from the 2nd to the 9th day. The mycelial growth index (MGI) was calculated using the expression MGI = Σ(D − Da)/N, where D is the actual mean colony diameter, Da is the previous mean diameter, and N is the number of growth days [38]. Based on the MGI, the lethal concentration that inhibited 50% of mycelial growth (EC50) of *C. truncatum* was calculated according to the methodology proposed by Torrez-Calzada et al. [40]. The experiment was conducted in a completely randomized design with 4 replications, each consisting of one Petri dish.

### 2.8. Activation of Biochemical Defense Mechanisms

Soybean seeds inoculated and treated with MnPhi and ZnPhi were subjected to the germination roll paper test, as described earlier. A total of 200 seeds per treatment were used, divided into eight replications, with 25 seeds per replication. The number of seeds used in this assay was lower than in the previous one to avoid root entangling. The seeds were incubated in a growth chamber at 25 ± 1 °C with a 12 h photoperiod at 40 μmol s^−1^ m^−2^. Plant material was collected at 24, 48, 72, 96, and 192 h after incubation (hai). The plant material was wrapped in aluminum foil and stored in a freezer at −80 °C until the beginning of the analyses. Three grams of plant material from each treatment was macerated in liquid nitrogen. An aliquot of 0.5 g and another of 30 mg were collected from each treatment for enzymatic analyses and lignin content determination, respectively. The 0.5 g aliquot was added to 2 mL microtubes, followed by the addition of 1.5 mL of 100 mM phosphate buffer, pH 7.8, and 1.5 mL of 0.1 mM EDTA and 10 mM ascorbic acid. The microtubes were centrifuged at 12,000× *g*, at 4 °C, for 15 min, and the supernatant was collected for enzymatic analyses. The protein concentration in the suspension was determined according to the Bradford [41] method, using bovine serum albumin as the standard, and adjusted for microquantities. For this, 10 µL of the sample, 20 µL of 100 mM potassium phosphate buffer, pH 7.8, and 170 µL of Bradford reagent were added to 96-well microplates. The absorbance was measured using a spectrophotometer (Biotek^®^, EUA) at 595 nm. A standard curve was prepared with bovine serum albumin (BSA) between 0 and 100 µg, and the results were expressed as the average of three replicates.

Peroxidase (POX) activity was evaluated using the guaiacol oxidation method, in which the enzyme mixture consisted of 30 μL of sample, 130 μL of 19.2 mM phosphate potassium buffer (pH 7), 20 μL of 50 mM guaiacol, and 20 μL of 125 mM hydrogen peroxide. The absorbance of the reaction mixture at 480 nm was measured after 10 min of incubation at 30 °C [42], and peroxidase activity was expressed as μmol of tetraguaiacol produced per minute per mg of protein. The molar extinction coefficient of 1.23 mM^−1^ cm^−1^ was used to calculate POX activity [43].

Catalase (CAT) activity was assessed by the decrease in absorbance at 240 nm for 3 min at 25 °C. The enzyme mixture consisted of 10 μL of the enzymatic extract, 60 μL of sterilized distilled water, 100 μL of 200 mM potassium phosphate buffer, pH 7, and 20 μL of 250 mM hydrogen peroxide. The molar extinction coefficient of 18 M^−1^ cm^−1^ was used to calculate the activity of this enzyme [44].

Superoxide dismutase (SOD) activity was determined by the enzyme’s ability to inhibit the photoreduction of nitro blue tetrazolium (NBT), as described by Giannopolitis and Ries [45]. In each well of a 96-well microplate, 10 μL of the enzymatic extract, 30 μL of sterilized distilled water, 2 μL of 0.2 mM riboflavin, 2 μL of 10 μM EDTA, 40 μL of 70 mM methionine, 100 μL of 100 mM potassium phosphate buffer, pH 7.8, and 15 μL of 1 mM NBT were added. The plates were incubated for 7 min in a closed chamber with a 30 W fluorescent lamp. Absorbance was measured at 560 nm, and the enzyme unit was defined as its ability to inhibit 50% of NBT photoreduction. Based on the activities of the CAT, POX, and SOD enzymes, the area under the activity progress curve (AUAPC) was calculated for CAT (AUAPCCat), POX (AUAPCPox), and SOD (AUAPCSod), following the methodology proposed by Shaner and Finney [46].

Soluble lignin was determined using the method proposed by Doster and Bostock [47]. A 30 mg sample of freeze-dried plant material was added to 1.5 mL of a solution of thioglycolic acid and hydrochloric acid (HCl) 2M (1:10 ratio). The samples were then incubated in a water bath for 4 h. Absorbance was measured at 280 nm in a spectrophotometer (PowerWave XS, Biotek^®^), and the value was expressed in μg.mg^−1^ dry weight. The evaluation of total soluble phenolic compounds was conducted according to the methodology of Spanos and Wrolstad [48]. Absorbance was measured at 725 nm in a spectrophotometer, and the amount was expressed in μg.mg^−1^ dry weight. The experiment was conducted in a completely randomized design.

### 2.9. Statistical Analysis

The experiments were conducted in duplicates, and the results were presented as the average of both experiments. Firstly, the assumptions of the analysis of variance were checked. To assess normality and homoscedasticity, the Shapiro–Wilk and Bartlett tests were used, respectively. The data did not follow the assumptions of the analysis of variance and needed to be transformed. In cases of significance, the data were transformed using √(x + 1). To accept or reject H0 (null hypothesis), the data were analyzed using the F test, and in case of significance, regression analysis or mean comparison tests were performed to differentiate the treatments. Scott-Knott, Tukey, and Dunnet tests (α < 0.05) were applied to determine the significance of the differences between the mean values. These analyses were carried out using the R program from the R Development Core Team [49]. The standard error was also calculated. The germination, health, and emergence data (transformed or not based on analysis 1) were differentiated using the Scott-Knott test. The biochemical and direct toxicity tests were conducted with Mn and Zn phosphites, which showed the best performance in the previous tests. In the biochemical tests, the means were differentiated using the Scott-Knott test. In the direct toxicity test, different doses of both phosphites (1.25, 2.50, 5.00, and 10.00 mL/L) were tested, with 5 mL/L corresponding to the dose used in previous tests. This assay included linear regression analysis (doses × MGSI) to determine the curves and calculate the dose with the highest MGSI inhibition. To compare the effect of these doses with the commercial fungicide, the Dunnett test (5%) was applied, which compares each dose individually with the fungicide.

## 3. Results

### 3.1. Effects of Phosphites on Seed Health and Germination

The test for the physiological and sanitary quality of the seeds showed that the seed lot had good sanitary quality, with less than 4% incidence of *C. truncatum* and a germination rate of 85.5% (Table 1). All the phosphites increased the germination of the inoculated seeds when compared to the inoculated and untreated seeds. Inoculated seeds treated with MnPhi and ZnPhi exhibited germination percentages similar to those of the non-inoculated seeds, and treatments with KPhi and CuPhi also increased the germination of the inoculated seeds but were less effective than MnPhi, ZnPhi, and the fungicide Protreat^®^. The phosphites also reduced the incidence of *C. truncatum* in the seeds. As with germination, MnPhi and ZnPhi did not differ from the fungicide Protreat^®^ and were the treatments that most reduced the incidence of *C. truncatum* in the inoculated seeds, followed by KPhi and CuPhi. The inoculated and untreated seeds had an incidence of *C. truncatum* above 96%.

### 3.2. Emergence and Development of Soybean Seedlings Under Greenhouse Conditions

*Colletotrichum truncatum* significantly reduced the emergence speed index (ESI), initial stand at 7 days (IS), and final stand (FS) of the inoculated and untreated seeds (Table 2). Consequently, there was lower plant height (H), root length (R), dry biomass of the aerial part (BA), and dry biomass of the roots (BR) of plants derived from these seeds. However, inoculated seeds treated with Phi and the fungicide showed superior results in all of these parameters compared to the inoculated, untreated seeds. Among the treatments, the highest ESI was observed when the inoculated seeds were treated with ZnPhi, MnPhi, and fungicide, followed by KPhi and CuPhi. Seven days after sowing, the treatments that showed the highest IS were ZnPhi and fungicide, followed by MnPhi and KPhi. Among the Phi, CuPhi had the lowest IS. Fourteen days after sowing, MnPhi, ZnPhi, and the fungicide achieved the highest FS, followed by KPhi. As with EI, CuPhi was the phosphite that showed the lowest FS, but it was still superior to the inoculated, untreated seeds. Regarding plant height, it was observed that MnPhi, KPhi, ZnPhi, and the fungicide induced the greatest plant growth, surpassing the plants derived from non-inoculated, untreated seeds, which did not differ from the seeds treated with CuPhi. The roots of plants derived from inoculated seeds treated with MnPhi, ZnPhi, and the fungicide exhibited greater growth, not differing from the roots of plants derived from non-inoculated seeds, followed by the treatments based on KPhi and CuPhi. Regarding BA, ZnPhi, KPhi, MnPhi, and the fungicide showed the best results, not differing from the non-inoculated seeds, followed by CuPhi. As for BR, the treatments that achieved the best results were MnPhi, ZnPhi, and fungicide, followed by CuPhi and KPhi, which were lower than those of the non-inoculated seeds.

### 3.3. Fungitoxic Activity of Phosphites

**Figure 1:**
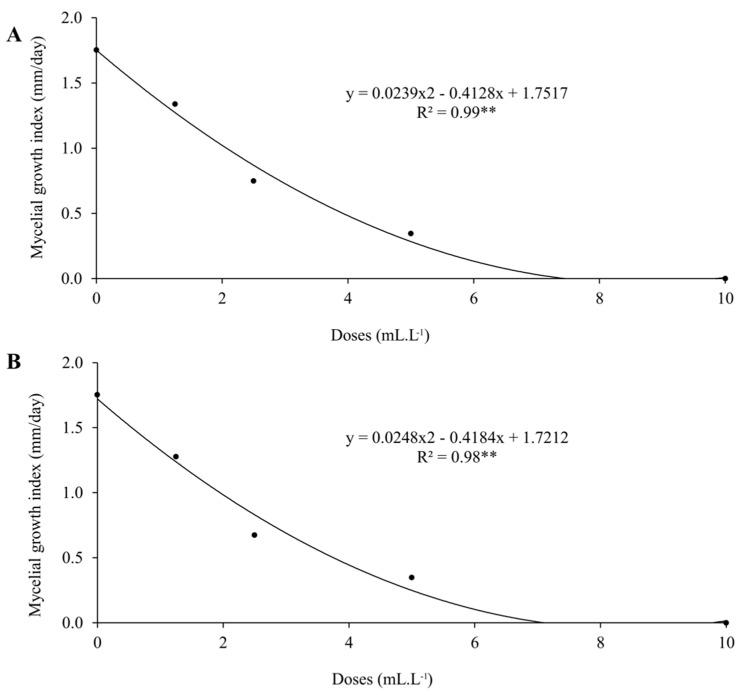
Doses of manganese (**A**) and zinc (**B**) phosphites on the mycelial growth index (mm/day) of *Colletotrichum truncatum*. ** Quality of fit of the regression model to the data.

**Figure 2:**
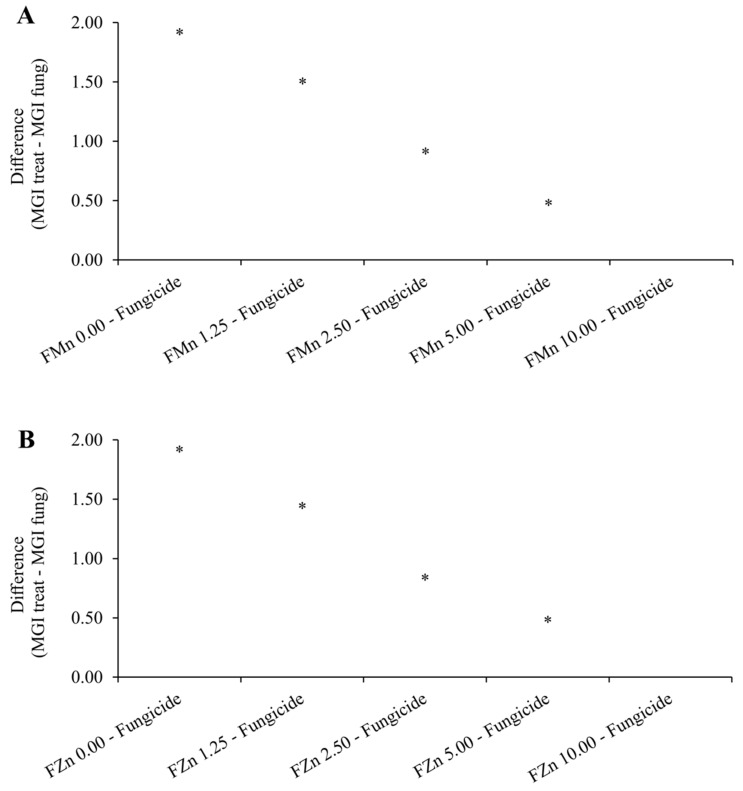
Dunnett test comparing the tested doses of Mn phosphite (**A**) and Zn phosphite (**B**) with the fungicide carbendazim + thiram. * There is a statistically significant difference between a treatment mean and the control group at a 5% significance level.

ZnPhi and MnPhi reduced the mycelial growth of *C. truncatum* with increasing doses (Figure 1), demonstrating that both have fungitoxic activity against the fungus. The DL50 and MIC of ZnPhi and MnPhi against *C. truncatum* were very similar, corresponding to a DL50 of 2.35 mL.L^−1^ (0.235 g.L^−1^ Zn) and 2.48 mL.L^−1^ (0.248 g.L^−1^ Mn), and MICs of 8.44 mL.L^−1^ (0.844 g.L^−1^ Zn) and 8.64 mL.L^−1^ (0.864 g.L^−1^ Zn), respectively (Table 3). At a dose of 8.6 mL.L^−1^ (0.86 g.L^−1^ Zn or Mn), the phosphites completely inhibited the mycelial growth of the fungus. At a dose of 10 mL.L^−1^ (1 g.L^−1^ Zn or Mn) (2 × the dose used in seed treatment), it would correspond to the dose of the fungicide carbendazim + thiram used for soybean seed treatment to control *C. truncatum* (Figure 2).

### 3.4. Activation of Biochemical Defense Mechanisms

**Figure 3:**
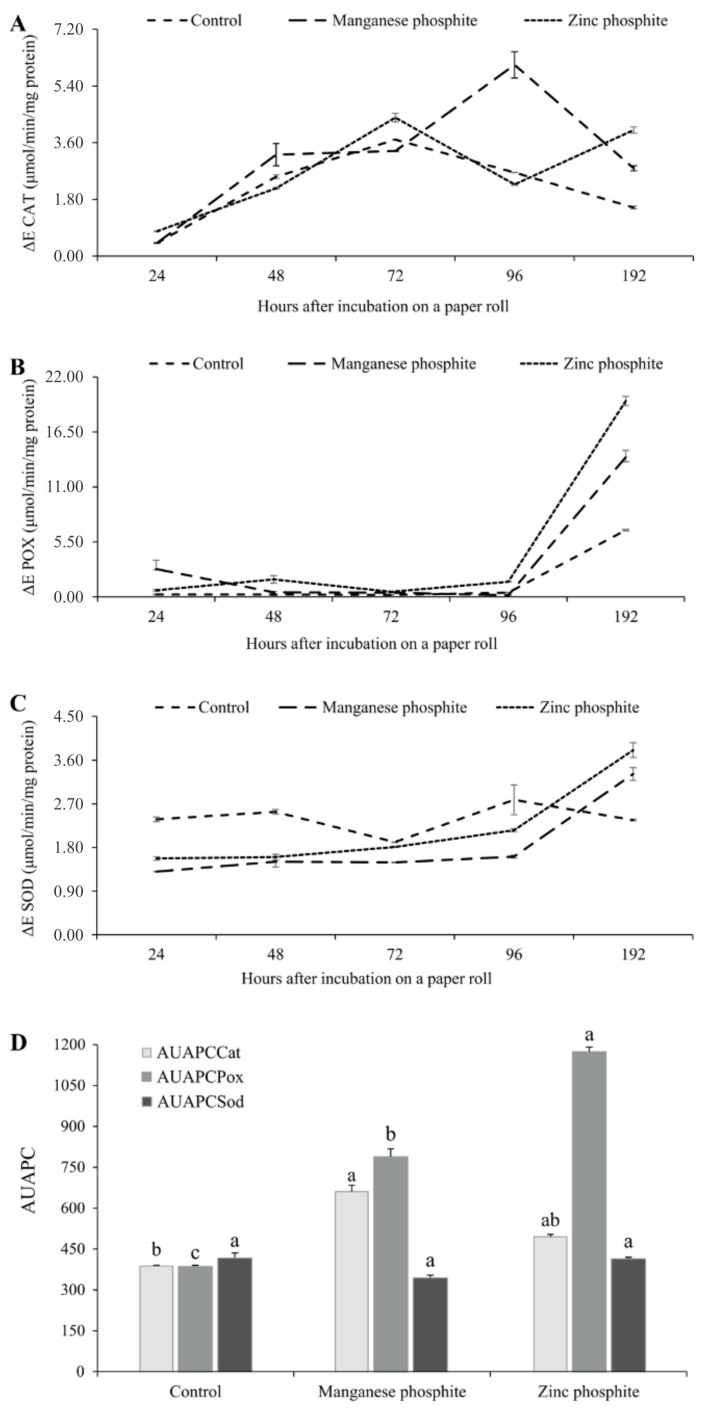
Activation of defense-related enzymes in soybean seeds inoculated with *Colletotrichum truncatum* and treated with Mn and Zn phosphite after incubation in paper rolls. (**A**) Catalase (CAT) activity, (**B**) Peroxidase (POX) activity, and (**C**) Superoxide dismutase (SOD) activity over 192 h, and (**D**) area under the progress curve for catalase (AUAPCCat), peroxidase (AUAPCPox), and superoxide dismutase (AUAPCSod). Columns with the same letter above did not differ according to the Tukey test, *p* ≤ 0.05.

An increase in the activity of the CAT and POX enzymes in soybean seeds infected with *C. truncatum* and treated with MnPhi and ZnPhi was observed (Figure 3). The increase in CAT activity was observed over time, with two activation peaks at 72 and 196 h after incubation when treated with ZnPhi, and at 96 h after incubation when treated with MnPhi (Figure 3A). No difference in the AUAPCCat at was observed in seeds treated with both Phi (Figure 3D). For the POX and SOD enzymes, activation peaks occurred at 192 h after incubation for seeds treated with both Phi (Figure 3B,C). However, seeds treated with ZnPhi presented higher AUAPCPox compared to seeds treated with MnPhi (Figure 3D). For AUAPCSod, no significant difference was found between MnPhi and ZnPhi, nor in relation to the control treatment (Figure 3D).

**Figure 4:**
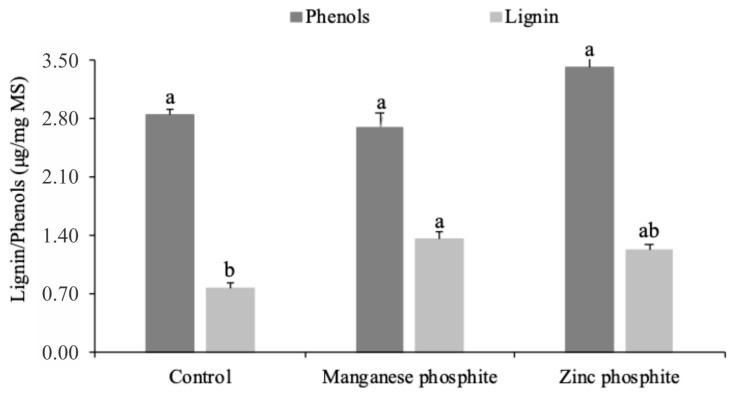
Total soluble phenolic content and soluble lignin content in seeds infected with *Colletotrichum truncatum* and treated with Zn and Mn phosphite. Columns with the same letter above did not differ according to the Tukey test, *p* ≤ 0.05.

No increase in the total soluble phenolic content was observed in the seeds treated with both phosphites (Figure 4). However, a significant increase in the lignin content was observed in the seeds treated with MnPhi.

## 4. Discussion

New products for soybean seed treatment to control *C. truncatum* are critically needed due to the increasing occurrence of anthracnose in productive fields in recent years and the resistance of the fungus to active ingredients of fungicides widely used in the main soybean growing areas worldwide. Additionally, there is a global trend toward seeking products that cause less environmental and public health impact and, in the case of soybeans, products that do not negatively interfere with nodulation. The irrational use of synthetic pesticides in soybean plantations has caused serious environmental problems, such as reduced biodiversity in agroecosystems and reduced nodulation by nitrogen-fixing bacteria, such as those of the *Bradyrhizobium* genera [18,22]. However, in recent decades, considerable evidence has been provided to establish the value of phosphites (Phi) in suppressing a range of plant diseases [21]. Despite Yáñez-Juárez et al. [22] reporting that phosphites are generally less effective in reducing damage caused by plant pathogens and could not completely replace fungicides, we found that ZnPhi and MnPhi were as effective as the commercial fungicide in controlling anthracnose in soybeans. Similarly, CuPhi, ZnPhi, and K/MnPhi phosphites were also more effective than the fungicide in controlling powdery mildew in eucalyptus seedlings [24]. Soybean seeds treated with MnPhi were more efficient than the commercial fungicide based on fludioxonil + metalaxyl in controlling damping-off caused by *Pythium aphanidermatum*, *P. irregulare*, and *P. ultimum* [21]. Several commercial products containing Phi are available on the market. Most formulations vary in composition, properties, application rates, and the type of cation bound to the Phi molecule [21]. Phi has the advantages of low cost and excellent absorption by plants, being easily transported within plants and displaying the unique property of moving through the plant not only via xylem but also via phloem [32,33]. Due to their efficient and rapid translocation in plant tissue, they can be applied to the canopy, stems, roots, or fruits [22], accumulate in regions of rapid growth, such as roots and shoots [34] and, because they are not metabolized by the plants [32,50], they can remain there for months or even years, preventing pathogen attacks [51,52]. These characteristics bring important advantages when considering soybean seed treatment for controlling anthracnose, which occurs both in seeds and in the aerial part of the plant. In addition to possessing protective and curative properties [53], Phi has shown low phytotoxicity [21,27], stimulates arbuscular mycorrhizal colonization [54,55], and can be mixed with other defense inducers, biological control agents, or fungicide molecules without causing inactivation or reduction in the activity of biocontrol agents [30,56]. The application of MnPhi in association with *Pseudomonas fluorescens* in soybean seed treatment significantly reduced charcoal root rot (*Macrophomina phaseolina*) [30]. A reduction in the number of *Meloidogyne javanica* eggs in soybean roots treated with MnPhi was also observed [57]. These results demonstrate that MnPhi can be used in soybean seed treatment for controlling various plant pathogens.

MnPhi and ZnPhi caused direct toxicity to *C. truncatum*, inhibiting its growth in culture media, promoting control of anthracnose in soybean seeds, stimulating seedling growth, and inducing catalase (CAT) and peroxidase (POX) enzyme activity, as well as promoting increases in lignin content in plant tissues, in the case of MnPhi. Different studies have also demonstrated the direct action of phosphites that limits the growth, development, and reproduction of plant pathogens. This is due to a combination of factors, such as the formation of pores and lysis of the fungal cell wall and membrane, leading to the efflux of cellular content [24,58] and transcriptional changes in several genes that code for proteins involved in the biosynthesis of cell wall components, amino acid synthesis, protein metabolism, and oxidative stress [19,59]. The decrease in the pH of the culture medium is also a factor that has led to the reduction of microorganism growth [39,60]. The level of growth reduction is determined by the tested organism, the amount of phosphite added, the pH produced in the culture medium, and the type of ion bound to the phosphite [39,60]. It is likely that the level of resistance of pathogens to phosphites is minimal because phosphites act on various targets within the pathogens [51,61]. However, there have been reports of resistance in isolates, such as *Bremia lactucae* in lettuce [62] and *P. cinnamomi* in avocados [63]. Therefore, the use of phosphites should be implemented within an integrated management program.

These changes and the rupture of fungal structures can lead to the release of elicitor molecules, triggering plant defense mechanisms [59,64]. The ability to induce resistance in plants when treating them with phosphites has also been documented. Biochemical and structural defense mechanisms, such as the production of pathogenesis-related proteins, phytoalexins, reactive oxygen species, deposition of callose, lignin, and suberin that restrict pathogen penetration and survival in the plant, have been observed when the phosphite ion enters plant tissue cells [22,65,66]. We observed that seedlings originated from seeds treated with MnPhi had increases in CAT and POX activities, as well as increases in lignin content. ZnPhi also promoted significant increases in CAT and POX activities. The activation of CAT and POX enzymes is likely due to Mn and Zn acting as cofactors for these enzymes. According to Zhao et al. [67], increased Mn supply led to higher activity of SOD and CAT enzymes in *Phytolacca americana* plants, while increased Zn supply raised POX activity. Higher doses of Mn also promoted significant increases in CAT and POX activities in barley [68]. MnPhi induced higher activities of the enzymes ascorbate peroxidase (APX), CAT, SOD, and polyphenol oxidase (PPO) in coffee seedlings inoculated with *Hemileia vastatrix* [23]. Regarding lignin, Rengel et al. [69] found that increased Mn concentration in soil induced higher lignin content in wheat (*Triticum aestivum*) plants. These authors correlated the increased lignin content with plant resistance to the fungus *Geumannomyces graminis*. According to Dordas [70], Mn controls lignin and suberin biosynthesis by activating numerous enzymes in the shikimate and phenylpropanoid pathways, where peroxidase can act. In this study, both ZnPhi and MnPhi significantly increased POX activity. Peroxidase catalyzes the oxidation and eventual polymerization of hydroxycinnamic alcohol in the presence of hydrogen peroxide, resulting in lignin [71]. Therefore, the increase in lignin is related to higher POX activity. According to Lobato et al. [29], resistance-inducing products may involve a metabolic cost resulting from the production of defense compounds or structures. However, these authors observed that CaPhi and KPhi induced defense mechanisms in seed tubers without being detrimental to plant growth [29], which was also observed in our study. We observed that the defense response in soybean seedlings was rapidly activated. This may be due to the rapid absorption of the phosphites by the roots. Griffith, Coffey, and Grant [72] suggested that plant cells may absorb phosphites more quickly than phosphorus.

We observed that the phosphites, except CuPhi, induced greater growth of the aerial part and roots of soybean, whose seeds were previously treated. However, there is much controversy, as studies question the effects of phosphite on growth promotion [31,73], while others have shown their capacity to stimulate aerial parts and root growth, as observed in wheat, canola, sugarbeet, ryegrass and *Arabidopsis thaliana* [26,55,74]. Phosphite cannot be converted into phosphate, so it does not improve plant growth through a nutritional mechanism [31]. However, further studies are needed to elucidate these mechanisms.

Although KPhi and CuPhi showed intermediate results in treating soybean seeds infected with *C. truncatum*, some studies have demonstrated their effectiveness in controlling certain diseases and inducing resistance in plants, such as KPhi against *Phytophthora infestans* in potatoes [65], *Pythium* spp. in soybeans [21], CuPhi controlling powdery mildew in eucalyptus [24,58], and *Pythium* spp. in cucumbers [27].

This study presents sufficient data and evidence to show that ZnPhi and MnPhi can be used as an effective alternative in soybean seed treatments for controlling *C. truncatum*. The use of both Phi in soybean seed treatments has shown numerous benefits, such as efficient control of anthracnose in already-infected seeds; the ability to control various soybean diseases, as observed in other studies; the potential to protect the aerial parts against anthracnose due to their rapid absorption and translocation; the ability to stimulate soybean growth and induce resistance mechanisms; low cost; the amount of Phi required to treat seeds is much lower than what is needed for foliar application; and the use of these products constitutes a viable and sustainable method for effective soybean seed protection against anthracnose. As far as we are aware, this study is the first report of using Phi as a soybean seed treatment for controlling *C. truncatum*. Further studies are needed to evaluate the duration of protection provided by Phi, both in the aerial and root parts, and the spectrum of action against different diseases affecting soybean crops. This will allow for a better understanding of the performance of these products in disease management.

## 5. Conclusions

The results of this study demonstrate the potential of Mn and Zn phosphites in controlling *Colletotrichum truncatum* in soybean seeds. The application of these phosphites resulted in improved seed germination, plant vigor, and emergence, while also effectively reducing fungal incidence. These findings suggest that phosphites not only exhibit direct antifungal properties but may also induce defense mechanisms in soybean plants, enhancing their resistance to *C. truncatum*. The biochemical analyses confirmed that the phosphites triggered enzymatic activity associated with plant defense, such as peroxidase and catalase, indicating a possible induction of systemic resistance. Furthermore, the direct toxicity assay showed that phosphites at higher concentrations significantly inhibited fungal growth, reinforcing their potential as an alternative to conventional fungicides. The regression analysis allowed us to determine the most effective phosphite doses for inhibiting fungal development, providing valuable insights into their optimal use. When compared to a commercial fungicide, the phosphites exhibited promising results, supporting their potential application in integrated disease management strategies. In conclusion, Mn and Zn phosphites emerge as sustainable alternatives for soybean seed treatment, combining disease control with plant growth promotion. Future studies should focus on field trials and the long-term effects of phosphite application on plant health, yield, and environmental impact to further validate their practical application in commercial soybean production.

## Figures and Tables

**Table 1:** Percentage of germination and sanitary quality of soybean seeds non-inoculated and inoculated with *Colletotrichum truncatum* and treated with Cu, K, Zn, and Mn phosphites and with the fungicide Protreat^®^ (150 g of carbendazim.L^−1^ + 350 g of thiram.L^−1^).

Treatments	Germination (%)	Incidence of *C. truncatum* in the Seeds (%)	Reduction of *C. truncatum* Incidence in the Seeds (%)
Inoculated control	25 ± 1.0 ^d^*	96 ± 3.7 ^e^*	0
Uninoculated control	85 ± 3.4 ^a^	4 ± 4.4 ^a^	95
Protreat^®^ fungicide	83 ± 10.8 ^a^	27 ± 6.9 ^b^	72
Copper phosphite	69 ± 3.4 ^b^	70 ± 7.2 ^d^	27
Manganese phosphite	81 ± 3.4 ^a^	29 ± 5.7 ^b^	69
Potassium phosphite	59 ± 10.8 ^c^	49 ± 4.8 ^c^	49
Zinc phosphite	85 ± 5.2 ^a^	33 ± 4.1 ^b^	65

* Mean values ± standard errors obtained from eight replicates. The means followed by same lower-case letter in the column do not differ by the Scott Knott test, *p* ≤ 0.05.

**Table 2:** Emergence speed index (ESI), initial stand at 7 days (IS), final stand at 14 days (FS), plant height (H), root length (R), dry biomass of the aerial part (BA) and roots (BR) at 28 days, of seedlings derived from soybean seeds inoculated or not with *Colletotrichum truncatum*, subjected to different treatments based on Cu, K, Zn, and Mn phosphites and the fungicide Protreat^®^ (150 g of carbendazim.L^−1^ + 350 g of thiram.L^−1^).

Treatments	ESI *	IS * (%)	FS * (%)	H * (cm)	R * (cm)	BA * (g)	BR * (g)
Inoculated control	21.5 ± 1.2 ^d^*	32.2 ± 1.5 ^e^*	34.0 ± 4.1 ^e^*	24.3 ± 0.7 ^c^*	20.4 ± 2.5 ^c^*	2.9 ± 0.4 ^c^*	1.6 ± 0.1 ^d^*
Uninoculated control	65.7 ± 2.8 ^a^	93.0 ± 2.9 ^a^	95.0 ± 2.1 ^a^	28.9 ± 1.7 ^b^	32.8 ± 1.1 ^a^	7.5 ± 0.7 ^a^	5.1 ± 0.6 ^a^
Protreat^®^ fungicide	45.9 ± 3.2 ^b^	71.0 ± 3.5 ^b^	76.7 ± 5.6 ^b^	30.3 ± 0.7 ^a^	30.2 ± 3.0 ^a^	6.8 ± 0.7 ^a^	3.8 ± 0.7 ^b^
Copper phosphite	33.3 ± 5.4 ^c^	51.5 ± 7.0 ^d^	55.0 ± 9.6 ^d^	28.6 ± 1.0 ^b^	25.0 ± 2.1 ^b^	4.6 ± 0.6 ^b^	2.6 ± 0.8 ^c^
Manganese phosphite	44.1 ± 2.7 ^b^	62.5 ± 7.5 ^c^	80.2 ± 1.2 ^b^	30.5 ± 1.6 ^a^	30.2 ± 0.9 ^a^	7.0 ± 0.3 ^a^	4.2 ± 0.2 ^b^
Potassium phosphite	39.0 ± 5.8 ^c^	60.0 ± 8.2 ^c^	64.0 ± 6.9 ^c^	30.2 ± 2.5 ^a^	24.4 ± 4.0 ^b^	5.8 ± 0.97 ^a^	2.4 ± 0.3 ^c^
Zinc phosphite	47.7 ± 2.9 ^b^	75.7 ± 5.3 ^b^	80.0 ± 3.3 ^b^	31.9 ± 1.6 ^a^	30.5 ± 0.1 ^a^	6.5 ± 0.2 ^a^	3.7 ± 0.4 ^b^

* Mean values ± standard errors obtained from eight replicates. Means followed by same lower-case letter in the column do not differ by the Scott Knott test, *p* ≤ 0.05.

**Table 3:** Lethal concentration (EC_50_) of Mn and Zn phosphites that inhibited 50% of the mycelial growth of *Colletotrichum truncatum* and the minimum inhibitory concentration (MIC). Active ingredient (a.i.): Zn or Mn.

Treatments	EC_50_ mL.L^−1^ (g.L^−1^ a.i.)	MCI mL.L^−1^ (g.L^−1^ a.i.)
Manganese phosphite	2.48 (0.248)	8.64 (0.864)
Zinc phosphite	2.35 (0.235)	8.44 (0.844)

## Data Availability

The datasets generated and/or analyzed during the current study are available in the Zenodo repository at https://doi.org/10.5281/zenodo.14787619.
